# Supplementary material for: A Variety of Mouse PYHIN Proteins Restrict Murine and Human Retroviruses
Source: Viruses. 2024 Mar 23;16(4):493. doi: 10.3390/v16040493 (PMC11054388; doi:10.3390/v16040493)
Supplement: Supplementary file 1 [file viruses-16-00493-s001.zip › viruses-2895539-supplementary.pdf]

A Variety of Mouse PYHIN Proteins Restrict Murine and Human Retroviruses

Sümeyye Erdemci-Evin, Matteo Bosso, Veronika Krchlikova, Wibke Bayer, Kerstin Regensburger, Martha Mayer, Ulf Dittmer, Daniel Sauter, Dorota Kmiec, Frank Kirchhoff

Supplementary material

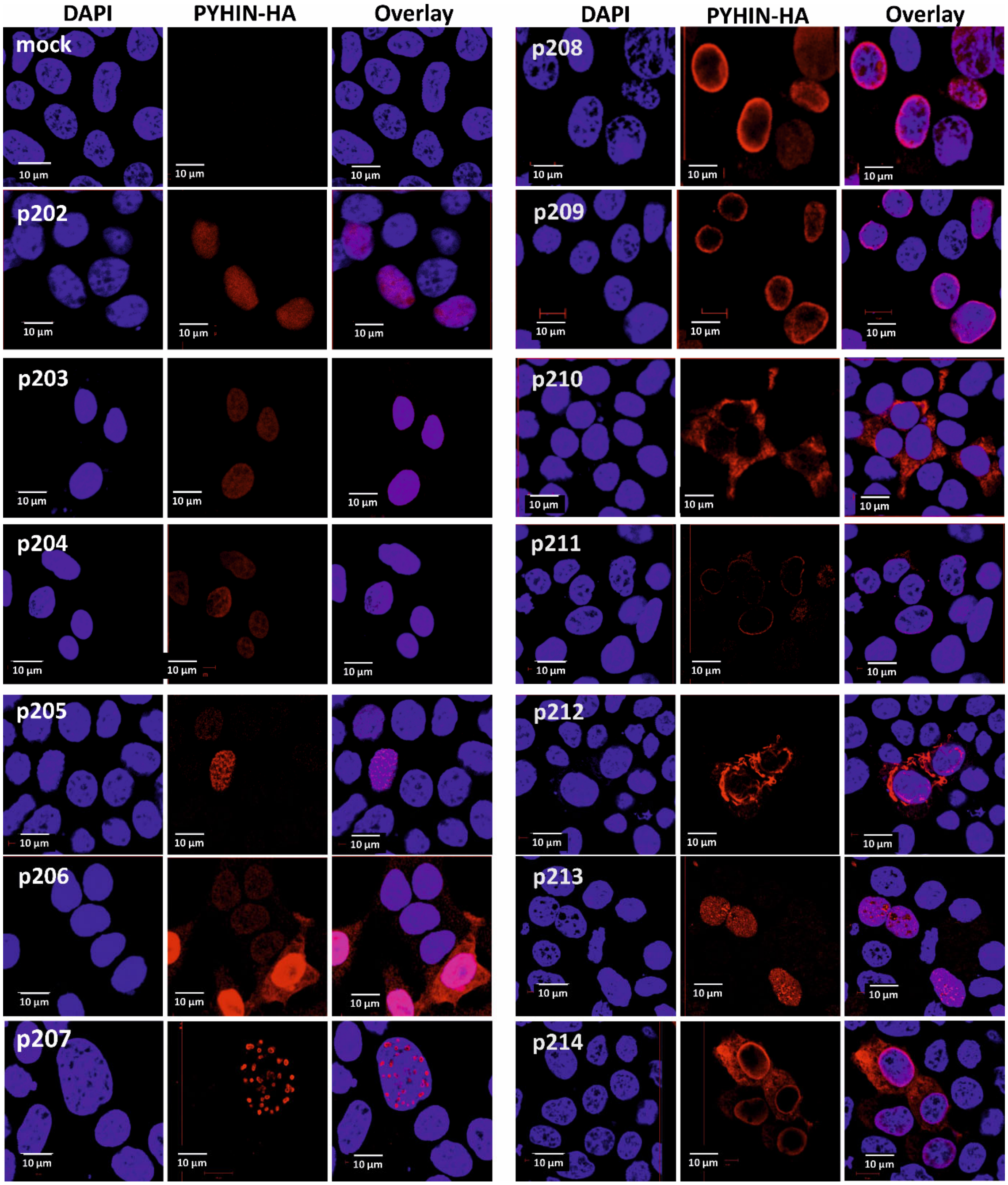

Supplementary Figure S1. Subcellular localization of murine PYHIN proteins. HEK293T cells were transfected with expression vectors for murine PYHIN proteins with a C-terminal HA-tag. 40h post-transfection, cells were fixed and stained with antibody targeting HA-tag to visualize PYHIN proteins (red) and DAPI to image the nuclei (blue).

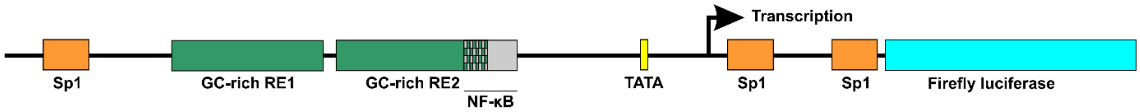

100
AATGAAAGACCCCACCAAATTGCTTAGCCTGATAGCCGCAGTAACGCCATTTTGCAAGGCATGGAAAAATACCAAACCAAGAATAGAGAAGTTCAGATCA
200
AGGGCGGGTACACGAAAACAGCTAACGTTGGGCCAAACAGGATATCTGCGGTGAGCAGTTTCGGCCCCGGCCCGGGGCCAAGAACAGATGGTCACCGCGG
300
TTCGGCCCCGGCCCGGGGCCAAGAACAGATGGTCCCCAGATATGGCCCAACCCTCAGCAGTTTCTTAAGACCCATCAGATGTTTCCAGGCTCCCCCAAGG
400
ACCTGAAATGACCCTGTGCCTTATTTGAATTAACCAATCAGCCTGCTTCTCGCTTCTGTTCGCGCGCTTCTGCTTCCCGAGCTCTATAAAAGAGCTCACA
500
ACCCCTCACTCGGCGCGCCAGTCCTCCGATAGACTGAGTCGCCCGGGTACCCGTGTATCCAATAAATCCTCTTGCTGTTGCATCCGACTCGTGGTCTCGC
558
TGTTCCTTGGGAGGGTCTCCTCAGAGTGATTGACTACCCGTCTCGGGGGTCTTTCATT

Supplementary Figure S2. Schematic representation of the F-MLV LTR luciferase construct and the viral LTR. Sp1/Sp3 and NF-κB binding sites were identified using the PROMO prediction tool and are indicated in the schematic and the LTR sequence.
